# 4-(9,10-Dioxo-9,10-dihydro­anthracen-1-yl)-4-oxobutanoic acid

**DOI:** 10.1107/S160053681004732X

**Published:** 2010-11-20

**Authors:** Yi Li, Qi-Sheng Lu, Rong-Qing Wei, Xiao-Ning Liu, Fang-Shi Li

**Affiliations:** a College of Biotechnology, and Pharmaceutical Engineering, Nanjing University of Technology, Nanjing 210009, People’s Republic of China; b Department of Applied Chemistry, College of Science, Nanjing University of Technology, Nanjing 210009, People’s Republic of China

## Abstract

In the title compound, C_18_H_12_O_5_, the anthracene moiety is almost planar (r.m.s. deviation = 0.0399 Å). In the crystal, mol­ecules are linked to each other by inter­molecular O—H⋯O and weak C—H⋯O hydrogen bonds.

## Related literature

For bond-length data, see: Allen *et al.* (1987 ▶). For applications of natural and synthetic anthraquinones, see: Brown (1980 ▶). For their activity, see: Johnson *et al.* (1997 ▶). For the synthesis, see: Inbasekaran *et al.* (1980 ▶).
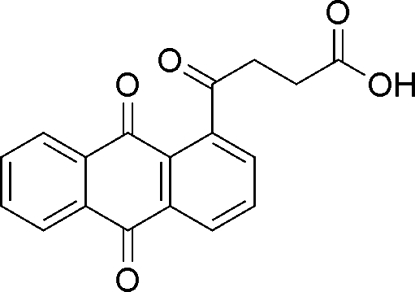


## Experimental

### 

#### Crystal data

C_18_H_12_O_5_
*M* *_r_* = 308.28
Monoclinic, 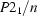

*a* = 5.168 (1) Å
*b* = 19.523 (4) Å
*c* = 14.367 (3) Å
β = 99.58 (3)°
*V* = 1429.3 (5) Å^3^
*Z* = 4
Mo *K*α radiation
μ = 0.11 mm^−1^
*T* = 293 K
0.20 × 0.10 × 0.10 mm

#### Data collection

Enraf–Nonius CAD-4 diffractometer
Absorption correction: ψ scan (North *et al.*, 1968 ▶) *T*_min_ = 0.979, *T*_max_ = 0.990
2892 measured reflections
2593 independent reflections
1048 reflections with *I* > 2σ(*I*)
*R*_int_ = 0.077
3 standard reflections every 200 reflections  intensity decay: 1%

#### Refinement

*R*[*F* ^2^ > 2σ(*F* ^2^)] = 0.078
*wR*(*F* ^2^) = 0.161
*S* = 1.00
2593 reflections
208 parameters
H-atom parameters constrained
Δρ_max_ = 0.21 e Å^−3^
Δρ_min_ = −0.23 e Å^−3^

### 

Data collection: *CAD-4 Software* (Enraf–Nonius, 1985 ▶); cell refinement: *CAD-4 Software*; data reduction: *XCAD4* (Harms & Wocadlo, 1995 ▶); program(s) used to solve structure: *SHELXS97* (Sheldrick, 2008 ▶); program(s) used to refine structure: *SHELXL97* (Sheldrick, 2008 ▶); molecular graphics: *SHELXTL* (Sheldrick, 2008 ▶); software used to prepare material for publication: *SHELXTL*.

## Supplementary Material

Crystal structure: contains datablocks I, global. DOI: 10.1107/S160053681004732X/bq2254sup1.cif

Structure factors: contains datablocks I. DOI: 10.1107/S160053681004732X/bq2254Isup2.hkl

Additional supplementary materials:  crystallographic information; 3D view; checkCIF report

## Figures and Tables

**Table 1:** Hydrogen-bond geometry (Å, °)

*D*—H⋯*A*	*D*—H	H⋯*A*	*D*⋯*A*	*D*—H⋯*A*
O5—H5*A*⋯O4^i^	0.82	1.86	2.681 (6)	177
C7—H7*A*⋯O1^ii^	0.93	2.43	3.255 (7)	147
C16—H16*A*⋯O3^iii^	0.97	2.48	3.375 (6)	154

Symmetry codes: (i) 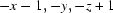; (ii) 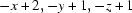; (iii) 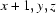.

**Fig. 1.**
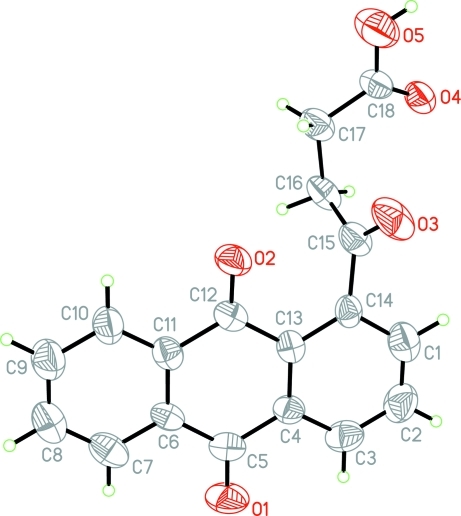
A drawing of the title molecular structure, with the atom-numbering scheme. Displacement ellipsoids are drawn at the 50% probability level.

**Fig. 2.**
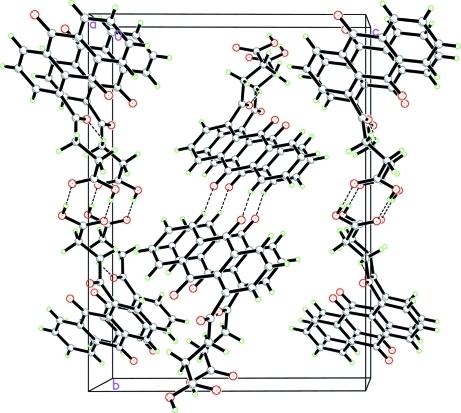
A packing diagram for (I). O—H···O and C—H···O intermolecular hydrogen bonds are shown by dashed lines.

## supplementary crystallographic information

### Comment

Anthraquinone compounds are widely used in the chemical industry and medicine. Natural and synthetic anthraquinone compounds are used in food, cosmetics, hair color agent and textile dyes (Brown *et al.*, 1980). In medicine, many of anthraquinones have diarrhea, anti-cell and other effects. The activity of anthraquinone derivatives has a great relationship with their planar frame structure (Johnson *et al.*, 1997). We report here the crystal structure of the title compound, (I).

The molecular structure of (I) is shown in Fig. 1. The bond lengths and angles are within normal ranges (Allen *et al.*, 1987). The anthrecene moiety is almost planar with an r.m.s. deviation of 0.0399 Å and a maximum deviation of 0.099 (4) Å for O2. In the crystal, molecules are linked to each other to form chains framework *via* intermolecular O—H···O and weak C—H···O hydrogen bonds.

### Experimental

The compound 4-(anthracen-1-yl)butanoic acid was synthesized by the method (Inbasekaran *et al.*, 1980). The crystals of the title compound (I) were obtained by dissolving the compound 4-(anthracen-1-yl)butanoic acid in methanol (25 ml) in the presence of oxygen and evaporating the solvent slowly at room temperature for about 10 d.

### Refinement

H atoms were positioned geometrically, with O—H = 0.82Å (for OH) and C—H = 0.93 and 0.97Å for aromatic and methylene H, respectively, and constrained to ride on their parent atoms, with *U*_iso_(H) = *xU*_eq_(C/N), where *x* = 1.2 for aromatic H.

### Crystal data

**Table:**

C_18_H_12_O_5_	*F*(000) = 640
*M**_r_* = 308.28	*D*_x_ = 1.433 Mg m^−^^3^
Monoclinic, *P*2_1_/*n*	Mo *K*α radiation, λ = 0.71073 Å
Hall symbol: -P 2yn	Cell parameters from 25 reflections
*a* = 5.168 (1) Å	θ = 8–12°
*b* = 19.523 (4) Å	µ = 0.11 mm^−^^1^
*c* = 14.367 (3) Å	*T* = 293 K
β = 99.58 (3)°	Needle, colourless
*V* = 1429.3 (5) Å^3^	0.20 × 0.10 × 0.10 mm
*Z* = 4	

### Data collection

**Table:**

Enraf–Nonius CAD-4 diffractometer	1048 reflections with *I* > 2σ(*I*)
Radiation source: fine-focus sealed tube	*R*_int_ = 0.077
graphite	θ_max_ = 25.3°, θ_min_ = 1.8°
ω/2θ scans	*h* = 0→6
Absorption correction: ψ scan (North *et al.*, 1968)	*k* = 0→23
*T*_min_ = 0.979, *T*_max_ = 0.990	*l* = −17→17
2892 measured reflections	3 standard reflections every 200 reflections
2593 independent reflections	intensity decay: 1%

### Refinement

**Table:**

Refinement on *F*^2^	Primary atom site location: structure-invariant direct methods
Least-squares matrix: full	Secondary atom site location: difference Fourier map
*R*[*F*^2^ > 2σ(*F*^2^)] = 0.078	Hydrogen site location: inferred from neighbouring sites
*wR*(*F*^2^) = 0.161	H-atom parameters constrained
*S* = 1.00	*w* = 1/[σ^2^(*F*_o_^2^) + (0.040*P*)^2^] where *P* = (*F*_o_^2^ + 2*F*_c_^2^)/3
2593 reflections	(Δ/σ)_max_ < 0.001
208 parameters	Δρ_max_ = 0.21 e Å^−^^3^
0 restraints	Δρ_min_ = −0.23 e Å^−^^3^

### Special details

**Table:**

Geometry. All e.s.d.'s (except the e.s.d. in the dihedral angle between two l.s. planes) are estimated using the full covariance matrix. The cell e.s.d.'s are taken into account individually in the estimation of e.s.d.'s in distances, angles and torsion angles; correlations between e.s.d.'s in cell parameters are only used when they are defined by crystal symmetry. An approximate (isotropic) treatment of cell e.s.d.'s is used for estimating e.s.d.'s involving l.s. planes.
Refinement. Refinement of *F*^2^ against ALL reflections. The weighted *R*-factor *wR* and goodness of fit *S* are based on *F*^2^, conventional *R*-factors *R* are based on *F*, with *F* set to zero for negative *F*^2^. The threshold expression of *F*^2^ > σ(*F*^2^) is used only for calculating *R*-factors(gt) *etc*. and is not relevant to the choice of reflections for refinement. *R*-factors based on *F*^2^ are statistically about twice as large as those based on *F*, and *R*- factors based on ALL data will be even larger.

### Fractional atomic coordinates and isotropic or equivalent isotropic displacement parameters (Å^2^)

**Table:**

	*x*	*y*	*z*	*U*_iso_*/*U*_eq_	
O1	0.6562 (8)	0.45510 (18)	0.4314 (3)	0.0687 (12)	
O2	0.2523 (6)	0.26823 (17)	0.6393 (2)	0.0534 (10)	
O3	−0.2936 (7)	0.21707 (19)	0.5131 (3)	0.0722 (13)	
O4	−0.2825 (7)	0.04974 (17)	0.4635 (3)	0.0580 (11)	
O5	−0.3291 (8)	0.0347 (2)	0.6115 (3)	0.0796 (13)	
H5A	−0.4460	0.0090	0.5866	0.119*	
C1	−0.0859 (11)	0.2918 (3)	0.3566 (4)	0.0590 (16)	
H1A	−0.2305	0.2662	0.3293	0.071*	
C2	0.0084 (12)	0.3422 (3)	0.3052 (4)	0.0656 (17)	
H2A	−0.0687	0.3499	0.2429	0.079*	
C3	0.2165 (11)	0.3814 (3)	0.3457 (4)	0.0556 (15)	
H3A	0.2776	0.4161	0.3107	0.067*	
C4	0.3375 (10)	0.3703 (2)	0.4378 (4)	0.0412 (13)	
C5	0.5697 (10)	0.4127 (3)	0.4790 (4)	0.0467 (14)	
C6	0.6852 (10)	0.4001 (2)	0.5783 (3)	0.0408 (13)	
C7	0.9026 (11)	0.4383 (3)	0.6188 (4)	0.0555 (15)	
H7A	0.9726	0.4712	0.5832	0.067*	
C8	1.0149 (11)	0.4274 (3)	0.7122 (4)	0.0611 (17)	
H8A	1.1575	0.4537	0.7397	0.073*	
C9	0.9160 (11)	0.3780 (3)	0.7640 (4)	0.0640 (17)	
H9A	0.9928	0.3702	0.8264	0.077*	
C10	0.7023 (10)	0.3397 (3)	0.7236 (4)	0.0548 (15)	
H10A	0.6372	0.3060	0.7593	0.066*	
C11	0.5839 (9)	0.3501 (2)	0.6323 (4)	0.0425 (13)	
C12	0.3528 (10)	0.3090 (2)	0.5912 (4)	0.0425 (13)	
C13	0.2415 (9)	0.3191 (2)	0.4900 (3)	0.0365 (12)	
C14	0.0322 (9)	0.2783 (2)	0.4497 (3)	0.0392 (12)	
C15	−0.0699 (10)	0.2173 (3)	0.4968 (4)	0.0484 (14)	
C16	0.0972 (9)	0.1546 (2)	0.5123 (4)	0.0502 (14)	
H16A	0.2779	0.1679	0.5340	0.060*	
H16B	0.0898	0.1305	0.4529	0.060*	
C17	0.0093 (10)	0.1069 (2)	0.5841 (4)	0.0526 (15)	
H17A	0.1567	0.0784	0.6109	0.063*	
H17B	−0.0394	0.1343	0.6348	0.063*	
C18	−0.2128 (10)	0.0622 (2)	0.5464 (4)	0.0493 (14)	

### Atomic displacement parameters (Å^2^)

**Table:**

	*U*^11^	*U*^22^	*U*^33^	*U*^12^	*U*^13^	*U*^23^
O1	0.081 (3)	0.059 (3)	0.071 (3)	−0.018 (2)	0.029 (2)	0.018 (2)
O2	0.061 (3)	0.053 (2)	0.048 (2)	−0.0200 (19)	0.012 (2)	0.0045 (18)
O3	0.035 (2)	0.068 (3)	0.119 (4)	−0.007 (2)	0.027 (2)	−0.005 (2)
O4	0.060 (3)	0.061 (2)	0.049 (2)	−0.026 (2)	−0.003 (2)	0.0015 (19)
O5	0.088 (3)	0.086 (3)	0.067 (3)	−0.036 (3)	0.021 (2)	−0.005 (2)
C1	0.049 (4)	0.060 (4)	0.065 (4)	0.000 (3)	0.001 (3)	−0.015 (3)
C2	0.077 (5)	0.069 (4)	0.044 (4)	0.003 (4)	−0.006 (3)	−0.002 (3)
C3	0.072 (4)	0.051 (4)	0.046 (4)	0.000 (3)	0.018 (3)	0.007 (3)
C4	0.043 (3)	0.041 (3)	0.041 (3)	−0.004 (3)	0.011 (3)	0.003 (2)
C5	0.054 (4)	0.038 (3)	0.052 (4)	0.000 (3)	0.019 (3)	0.002 (3)
C6	0.045 (3)	0.030 (3)	0.050 (3)	−0.004 (3)	0.014 (3)	−0.005 (3)
C7	0.053 (4)	0.046 (3)	0.073 (4)	−0.006 (3)	0.025 (3)	−0.014 (3)
C8	0.051 (4)	0.062 (4)	0.069 (4)	−0.005 (3)	0.006 (3)	−0.019 (3)
C9	0.070 (4)	0.068 (4)	0.051 (4)	−0.012 (4)	0.002 (3)	−0.011 (3)
C10	0.054 (4)	0.062 (4)	0.046 (4)	−0.010 (3)	0.000 (3)	−0.005 (3)
C11	0.038 (3)	0.039 (3)	0.050 (3)	0.002 (3)	0.006 (3)	−0.009 (3)
C12	0.041 (3)	0.037 (3)	0.052 (4)	−0.002 (3)	0.013 (3)	−0.003 (3)
C13	0.031 (3)	0.041 (3)	0.038 (3)	0.003 (2)	0.008 (2)	−0.009 (3)
C14	0.031 (3)	0.039 (3)	0.047 (3)	0.005 (3)	0.001 (3)	−0.006 (3)
C15	0.039 (3)	0.039 (3)	0.067 (4)	−0.003 (3)	0.008 (3)	−0.009 (3)
C16	0.038 (3)	0.044 (3)	0.071 (4)	−0.009 (3)	0.016 (3)	−0.013 (3)
C17	0.051 (3)	0.038 (3)	0.068 (4)	−0.007 (3)	0.008 (3)	−0.006 (3)
C18	0.048 (4)	0.038 (3)	0.061 (4)	−0.010 (3)	0.007 (3)	0.004 (3)

### Geometric parameters (Å, °)

**Table:**

O1—C5	1.206 (5)	C7—H7A	0.9300
O2—C12	1.225 (5)	C8—C9	1.368 (7)
O3—C15	1.218 (5)	C8—H8A	0.9300
O4—C18	1.210 (6)	C9—C10	1.379 (6)
O5—C18	1.308 (6)	C9—H9A	0.9300
O5—H5A	0.8200	C10—C11	1.367 (6)
C1—C2	1.367 (7)	C10—H10A	0.9300
C1—C14	1.400 (6)	C11—C12	1.478 (6)
C1—H1A	0.9300	C12—C13	1.484 (6)
C2—C3	1.368 (7)	C13—C14	1.389 (6)
C2—H2A	0.9300	C14—C15	1.508 (6)
C3—C4	1.383 (6)	C15—C16	1.494 (6)
C3—H3A	0.9300	C16—C17	1.513 (6)
C4—C13	1.391 (6)	C16—H16A	0.9700
C4—C5	1.496 (6)	C16—H16B	0.9700
C5—C6	1.472 (6)	C17—C18	1.472 (6)
C6—C7	1.393 (6)	C17—H17A	0.9700
C6—C11	1.402 (6)	C17—H17B	0.9700
C7—C8	1.388 (7)		
			
C18—O5—H5A	109.5	C10—C11—C6	119.1 (5)
C2—C1—C14	120.8 (5)	C10—C11—C12	120.4 (5)
C2—C1—H1A	119.6	C6—C11—C12	120.5 (5)
C14—C1—H1A	119.6	O2—C12—C11	121.1 (5)
C1—C2—C3	119.9 (5)	O2—C12—C13	120.5 (5)
C1—C2—H2A	120.0	C11—C12—C13	118.4 (4)
C3—C2—H2A	120.0	C14—C13—C4	120.7 (5)
C2—C3—C4	121.2 (5)	C14—C13—C12	118.7 (5)
C2—C3—H3A	119.4	C4—C13—C12	120.6 (5)
C4—C3—H3A	119.4	C13—C14—C1	118.4 (5)
C3—C4—C13	118.9 (5)	C13—C14—C15	124.9 (5)
C3—C4—C5	119.8 (5)	C1—C14—C15	116.6 (5)
C13—C4—C5	121.3 (5)	O3—C15—C16	120.8 (5)
O1—C5—C6	122.4 (5)	O3—C15—C14	120.3 (5)
O1—C5—C4	120.2 (5)	C16—C15—C14	118.6 (4)
C6—C5—C4	117.4 (5)	C15—C16—C17	112.0 (4)
C7—C6—C11	119.4 (5)	C15—C16—H16A	109.2
C7—C6—C5	118.9 (5)	C17—C16—H16A	109.2
C11—C6—C5	121.6 (5)	C15—C16—H16B	109.2
C8—C7—C6	120.0 (5)	C17—C16—H16B	109.2
C8—C7—H7A	120.0	H16A—C16—H16B	107.9
C6—C7—H7A	120.0	C18—C17—C16	114.8 (5)
C9—C8—C7	120.1 (6)	C18—C17—H17A	108.6
C9—C8—H8A	120.0	C16—C17—H17A	108.6
C7—C8—H8A	120.0	C18—C17—H17B	108.6
C8—C9—C10	119.9 (6)	C16—C17—H17B	108.6
C8—C9—H9A	120.1	H17A—C17—H17B	107.6
C10—C9—H9A	120.1	O4—C18—O5	121.6 (5)
C11—C10—C9	121.5 (6)	O4—C18—C17	124.5 (5)
C11—C10—H10A	119.2	O5—C18—C17	113.8 (5)
C9—C10—H10A	119.2		
			
C14—C1—C2—C3	−1.8 (8)	C10—C11—C12—C13	−176.1 (4)
C1—C2—C3—C4	1.1 (8)	C6—C11—C12—C13	3.6 (6)
C2—C3—C4—C13	−1.2 (8)	C3—C4—C13—C14	1.8 (7)
C2—C3—C4—C5	178.2 (5)	C5—C4—C13—C14	−177.5 (4)
C3—C4—C5—O1	−2.2 (7)	C3—C4—C13—C12	−175.4 (4)
C13—C4—C5—O1	177.1 (5)	C5—C4—C13—C12	5.3 (7)
C3—C4—C5—C6	178.1 (4)	O2—C12—C13—C14	−4.1 (7)
C13—C4—C5—C6	−2.6 (7)	C11—C12—C13—C14	177.0 (4)
O1—C5—C6—C7	−0.1 (7)	O2—C12—C13—C4	173.2 (4)
C4—C5—C6—C7	179.6 (4)	C11—C12—C13—C4	−5.8 (6)
O1—C5—C6—C11	−179.3 (5)	C4—C13—C14—C1	−2.5 (7)
C4—C5—C6—C11	0.4 (7)	C12—C13—C14—C1	174.8 (4)
C11—C6—C7—C8	−0.8 (7)	C4—C13—C14—C15	173.8 (4)
C5—C6—C7—C8	180.0 (5)	C12—C13—C14—C15	−8.9 (7)
C6—C7—C8—C9	1.5 (8)	C2—C1—C14—C13	2.4 (7)
C7—C8—C9—C10	−0.9 (8)	C2—C1—C14—C15	−174.2 (5)
C8—C9—C10—C11	−0.4 (8)	C13—C14—C15—O3	118.0 (6)
C9—C10—C11—C6	1.1 (8)	C1—C14—C15—O3	−65.7 (7)
C9—C10—C11—C12	−179.2 (5)	C13—C14—C15—C16	−69.1 (6)
C7—C6—C11—C10	−0.4 (7)	C1—C14—C15—C16	107.2 (5)
C5—C6—C11—C10	178.7 (5)	O3—C15—C16—C17	−24.0 (7)
C7—C6—C11—C12	179.8 (4)	C14—C15—C16—C17	163.1 (4)
C5—C6—C11—C12	−1.1 (7)	C15—C16—C17—C18	81.7 (5)
C10—C11—C12—O2	4.9 (7)	C16—C17—C18—O4	18.2 (7)
C6—C11—C12—O2	−175.3 (4)	C16—C17—C18—O5	−163.3 (4)

### Hydrogen-bond geometry (Å, °)

**Table:**

*D*—H···*A*	*D*—H	H···*A*	*D*···*A*	*D*—H···*A*
O5—H5A···O4^i^	0.82	1.86	2.681 (6)	177
C7—H7A···O1^ii^	0.93	2.43	3.255 (7)	147
C16—H16A···O3^iii^	0.97	2.48	3.375 (6)	154

Symmetry codes: (i) −*x*−1, −*y*, −*z*+1; (ii) −*x*+2, −*y*+1, −*z*+1; (iii) *x*+1, *y*, *z*.
